# New Year's revolution

**DOI:** 10.1098/rsob.180005

**Published:** 2018-01-31

**Authors:** David M. Glover

**Affiliations:** *Editor in Chief*

It is hard to believe that it is already time again to greet another New Year but once again, it is a good opportunity to take stock of how far *Open Biology* has come, where we are going, and whether we are on the right track. As scientific publishing goes through perhaps its most profound revolution because it was established over 350 years ago, these are questions that we need to keep very much in mind.

*Open Biology* was started to provide a route for the rapid publication of original research and review articles of the highest quality in the broad field of cellular and molecular biology. We wanted articles to be reviewed rapidly, fairly and constructively and published in Open Access format so that they should be freely available for everyone to see. Another of our aims was to see more cell and molecular biology published by the Royal Society as part of a deeply felt conviction that academic societies, charities and university publishing houses can most fairly represent scientists. This was in some ways a response to a feeling that commercial publishing houses had a stranglehold over the ‘top-end' publishing in this area and a growing frustration at the slow and often arbitrary decision-making processes that seemed to hold sway in many leading journals. Getting a toe-hold in this highly competitive publishing niche has brought its challenges but now, we are climbing slowly up the cliff face.

I like to think that the Royal Society has kept abreast of the changing times in publishing and that we have responded to the needs of the community by embracing the open access model, by encouraging authors to use preprint servers such as *Biorxiv*, by using a wide-range of metrics to assess the impact of our papers, and by publishing referees' reports and authors' responses. *Open Biology* also has a sister journal, *Open Science*, into which articles can be cascaded, not just from *Open Biology* but from any of the Society's journals, if they are thought not to fit exactly with the objectives of the originally targeted journal. However, our underlying principle is one long-established—that fair and rapid publication of original work can be achieved by an academic society using the traditional refereeing system to provide constructive evaluations of papers by editors who are practising scientists.

The steady gain in our impact has only been possible by getting active scientists to join our editorial board—and our editorial board really do have to work as editors! Last year, I was able to announce the addition of an invaluable cohort of new editorial board members from North America and China. This year, we have further expanded our board membership, being particularly careful to try and represent all our community and avoid unconscious bias. I would like to say thank you to all these scientists for their invaluable contributions to the journal. Thank you also to our growing network of referees and most importantly, thank you to all our authors for submitting their original research papers and lively review articles. Our steady flow of review articles enliven the pages of the journal and bring a wide range of perspectives on diverse, rapidly developing fields.

Finally, we are sad to say good-bye to one of our founding editors, Stewart Cole. Stewart has worked tirelessly for the journal and through his efforts we have attracted some excellent papers covering a wide range of microbiology. Thank you, Stewart, for helping us in this difficult part of our climb. We hope that in 2018 and beyond, we can continue to live up to the high standards that you have helped to set.

